# From local challenges to local solutions: Insights from the 2025 Diabetes Summit Innovation Showcase

**DOI:** 10.4102/phcfm.v18i2.5563

**Published:** 2026-07-21

**Authors:** Atiya Mosam, Patrick Ngassa Piotie, Kibachio J. Mwangi, Melanie Pienaar

**Affiliations:** 1 Mayibuye Health, Johannesburg, South Africa; 2 School of Public Health, University of the Witwatersrand, Johannesburg, South Africa; 3 School of Health Systems and Public Health, Faculty of Health Sciences, University of Pretoria, Pretoria, South Africa; 4 Department of Public Health, Medical School, Nelson Mandela University, Gqeberha, South Africa; 5 World Health Organization, Pretoria, South Africa; 6 School of Nursing, Faculty of Health Sciences, University of the Free State, Bloemfontein, South Africa

## Introduction

South Africa faces a rapidly escalating diabetes burden, characterised by rising prevalence, suboptimal control and persistent inequities in access to prevention, care and complication screening.^1,2,3^ Against this backdrop, the 2025 Diabetes Summit convened a diverse set of stakeholders under the theme *Innovate for Impact: Transforming the Future of Diabetes in South Africa*. A central feature of the Summit was the Innovation Showcase, which provided a platform for implementers, researchers, civil-society organisations and health-system actors to present innovations responding to real-world challenges across the diabetes care continuum.

In this context, the term ‘innovation’ is used deliberately to denote locally developed, contextually grounded ways of working that respond to real-world constraints within the South African health system, rather than the introduction of novel technologies alone.^4^ The innovations showcased were largely locally conceived and implemented, responding directly to South Africa’s health-system realities, workforce limitations, social determinants of health and community needs.

This conference report synthesises selected innovations presented during the Innovation Showcase, drawing on submitted abstracts and oral presentations. The report highlights cross-cutting themes, shared implementation lessons and their implications for strengthening diabetes prevention and care in South Africa. Collectively, the innovations demonstrate how locally driven, evidence-informed approaches can address systemic bottlenecks while aligning with national priorities, including the National Strategic Plan for the Prevention and Control of Non-Communicable Diseases (NCDs) (2022–2027).^5^

## Overview of the Innovation Showcase

The Innovation Showcase featured a diverse range of initiatives spanning primary health care strengthening, complication screening, community-based support, digital health and interprofessional education (IPE). The innovations were purposively identified and invited through targeted engagement with individuals and organisations known for their work in diabetes and NCD care in South Africa, ensuring a broad and representative selection of locally grounded, contextually relevant solutions.

These seven initiatives were implemented across diverse settings, including urban townships, rural districts and public-sector primary health care facilities. While varying in scope and scale, they shared a common emphasis on feasibility, scalability and responsiveness to health-system constraints.

Key areas of innovation included: (1) task-shifting and workforce empowerment in primary care, (2) technology-enabled screening and clinical decision support, (3) community-centred and peer-led models of care and (4) intersectoral and interprofessional approaches to service delivery and training.

## Strengthening primary health care through task-shifting and clinical support

Several showcased innovations directly addressed clinical inertia and workforce constraints in primary health care. The Tshwane Insulin Project (TIP), developed by the University of Pretoria Diabetes Research Centre, exemplified an implementation science approach to optimising insulin use among people with poorly controlled type 2 diabetes at the primary health care level.^6,7^ By training nurses to initiate and titrate insulin using structured algorithms, supported through tele-mentorship via a digital referral platform, TIP challenged long-standing barriers linked to doctor-dependent initiation of insulin.

The project demonstrated meaningful clinical impact over a 14-week follow-up period, with substantial improvements in glycaemic control and increased nurse confidence in managing complex diabetes cases. Importantly, TIP integrated community health workers to support home-based follow-up and adherence, reinforcing continuity of care beyond clinic walls. The model highlights the potential of task shifting, when combined with digital support and clear clinical governance, to strengthen chronic disease management in resource-constrained settings.

## Technology-enabled screening and early detection of complications

Innovation Showcase presentations also underscored the role of technology in expanding access to diabetes complication screening. Artificial intelligence-assisted diabetic retinopathy screening initiatives, implemented in underserved communities such as Khayelitsha, illustrated how point-of-care retinal imaging coupled with artificial intelligence can facilitate early detection and referral. These models reduce dependence on specialist availability and lower barriers to screening in high-risk populations.^8^

Complementary approaches, such as the EYEcuity screening model, demonstrated how portable diagnostic tools and streamlined referral pathways can be integrated into existing primary care workflows. Together, these innovations emphasised that technological solutions must be adapted for local populations, embedded within functional referral systems and supported by trained personnel to translate detection into timely treatment.

## Community-centred and peer-led models of care

Beyond facility-based interventions, several innovations highlighted the importance of community engagement and peer support in diabetes care.^9,10^ Civil-society-led initiatives, including those presented by Sweet Life and partners, focused on diabetes education, patient empowerment and narrative-driven advocacy. These approaches leveraged lived experience, digital storytelling and community networks to improve diabetes literacy and counter stigma.^11,12^

Similarly, community-based programmes presented by humanitarian and non-governmental organisations illustrated how integrated care models can reach marginalised populations, including migrants and people affected by humanitarian crises. These initiatives reinforced the value of trust, cultural competence and continuity in sustaining engagement with people living with diabetes.

## Interprofessional education and rural health system strengthening

The Innovation Showcase also featured educational innovations aimed at strengthening the future health workforce. The IPE rural platform implemented by the University of the Free State Faculty of Health Sciences demonstrated how undergraduate and postgraduate students from multiple disciplines can be trained collaboratively while delivering services in rural communities. Diabetes screening, home visits, lifestyle groups and referral coordination formed part of a broader engaged-scholarship model.

This approach addressed dual objectives: improving access to services in underserved rural areas and equipping future health professionals with competencies in teamwork, community engagement and person-centred care. The model highlights the potential of academic institutions to act as health-system partners in the NCD response, particularly in rural and peri-urban settings where workforce shortages are most acute.

## Cross-cutting themes and implementation lessons

Across the Innovation Showcase, several cross-cutting themes emerged. Firstly, innovation was largely pragmatic rather than technological. Successful initiatives were grounded in an understanding of health-system realities, including workforce limitations, fragmented referral pathways and social determinants of health.

Secondly, partnerships were central to implementation. Collaborations between academia, government, civil society and communities enabled shared ownership and facilitated scale-up discussions. Thirdly, many of the innovations demonstrated explicit alignment with national policy priorities, positioning them as potential candidates for adoption and scale-up. At the same time, participants acknowledged that resource mobilisation will remain a critical challenge for ensuring their scalability and long-term sustainability.

Fourthly, the importance of data and learning was evident. Whether through clinical outcomes, screening coverage or process indicators, innovators emphasised the need for monitoring and evaluation to inform adaptation and advocacy.

## Implications for diabetes policy and practice in South Africa

The innovations presented at the 2025 Diabetes Summit reinforce the argument that addressing South Africa’s diabetes burden requires more than clinical guidelines or action from the public sector alone. Scalable solutions must draw on the diverse stakeholders already innovating to improve diabetes prevention and care and should be embedded within a coherent national response that enables task shifting, supports digital health adoption, strengthens referral systems and recognises the value of community-based care.

The Innovation Showcase provided concrete examples that can inform the development of a National Diabetes Strategy, complementing existing NCD frameworks. By documenting and synthesising these innovations, this conference report contributes to the evidence base needed to advocate for policy reform, sustainable financing and multisectoral coordination.

## Conclusion

The Innovation Showcase at the 2025 Diabetes Summit demonstrated that impactful solutions to diabetes challenges already exist within South Africa. These innovations, rooted in local contexts and driven by committed practitioners and communities, offer practical pathways to improve prevention, care and outcomes. Capturing and disseminating these lessons is an important step toward ensuring that innovation translates into sustained system-wide impact.
